# Supplementary material for: Long-term consistency of personality traits of cattle
Source: R Soc Open Sci. 2020 Feb 12;7(2):191849. doi: 10.1098/rsos.191849 (PMC7062087; doi:10.1098/rsos.191849)
Supplement: Detailed methods and analysis [file rsos191849supp1.docx]

**Detailed Methods and Analysis (Supplementary Material) for:**

**Long-term consistency of personality traits of cattle**

Heather W. Neave, Joao H. C. Costa, Daniel M. Weary, and Marina A. G. von Keyserlingk

Corresponding author: Marina von Keyserlingk, email: nina@mail.ubc.ca

***Animal management***

This experiment was carried out between November 2014 to February 2018 at the University of British Columbia’s Dairy Education and Research Centre, located in Agassiz, British Columbia, Canada (49°N, 121°W). All procedures carried out in this study were approved by the UBC Animal Ethics Committee (protocol # A15-0117). The animals were cared for according to the guidelines outlined by the Canadian Council of Animal Care (2009).

This experiment used two cohorts of Holstein calves (Cohort 1: n = 33 female calves; Cohort 2: n = 32 female calves) that were studied longitudinally from pre-weaning to first lactation. Cohort 2 also contained n = 24 male calves that were studied from pre-weaning to post-weaning. All animals were reared on the same farm and management of animals was the same for all periods except for the pre-weaning period. Calves in Cohort 1 (born November 2014 to Feb 2015) received 12 L/d of milk for 6 wk then weaned over 2 wk to complete weaning at 8 wk ( pre-weaning period was not recorded for this Cohort), while calves in Cohort 2 received different milk allowances (6, 8, 10 or 12 L/d of milk) and weaned on a step-down weaning program reported in Neave et al. [1] (milk allowance did not affect measures of personality, see Neave et al. [1]). After the post-weaning period, all animals were managed according to standard farm management procedures and housed in free-stall pens of at least 12 animals with deep-bedded sand. Animals did not necessarily remain in the same social groups at all periods. Some females and all male calves were sold after weaning and thus were unavailable for final testing, resulting in 22 of the original 33 female calves in Cohort 1 and 26 of the original 32 female calves in Cohort 2 completing the study. However, the data from animals that did not complete the study were still used for any periods in which they were available.

***Personality tests***

Animals were subjected individually to 3 tests designed to measure behavioural reactivity to novelty at 4 different periods: pre-weaning (age 27 ± 3 d for Cohort 2: n = 56; Cohort 1 was not tested), post-weaning (age 100 ± 12 d for Cohort 1: n = 33; age 76 ± 3 d for Cohort 2: n = 56), around puberty (hereafter called ‘puberty’; age 11.7 ± 0.4 mo, Cohort 2: n = 30; Cohort 1 was not tested), and during first lactation after confirmation of second pregnancy (hereafter called ‘lactation’; age 29.6 ± 2.9 mo for Cohort 1: n = 22; age 29.6 ± 1.9 mo for Cohort 2: n = 26). Cohort 1 was not tested in the pre-weaning and puberty periods; this was because Cohort 1 was a convenience sample where researchers were only able to test in the post-weaning and lactation periods due to time constraints.

The personality tests conducted at each period included a novel environment, novel human and novel object test, following Neave et al. [1] and modified from Van Reenen et al. [2]. Animals were exposed individually to one test per day for 3 consecutive days in a pen that was visually isolated from people and other animals. Each testing pen was bedded with sawdust. Tests during the pre-weaning and post-weaning periods took place in an arena measuring 4.7 x 4.3 m that was identical to the home pen except that plywood was used to block access to the feeding equipment. Tests at puberty took place in an arena measuring 9.4 m x 4.8 m and all 4 walls were constructed of plywood. Tests during lactation took place in an arena measuring 12.0 m x 12.0 m. Three of the walls were made of concrete and the fourth wall was constructed of black heavy-duty plastic suspended from the ceiling to the floor, achieving visual isolation from other cows.

For all tests, each animal was guided gently out of the home pen by two familiar people and into the test pen. Behaviors during each test were recorded continuously using one camera (WV-CW504SP, Panasonic, Osaka, Japan) positioned 8 m above the test arena. A single observer scored all behaviors in all tests following Neave et al. [1] (see Supplementary Table S1) after achieving sufficient inter-observer reliability (Cohen’s kappa κW > 0.80). The test started when the animal had all four feet inside the arena. Animals that never touched the human or object were assigned a maximum latency of 10 or 15 min for the human and object tests, respectively.

For the novel environment test, animals entered the empty arena individually and remained there for 30 min. On the recorded video, four quadrants were superimposed over the arena image and the number of crossings between zones was recorded. Additional behavioral measures included time spent with nose contact to the walls and floor (including licking and sniffing), time spent lying down, time spent inactive, and the number of withdrawals, bucking and locomotory play events.

For the novel human test, animals entered the arena individually for 10 min where an unfamiliar person wearing clean coveralls was standing immobile in the centre of the arena. The person kept arms and hands close to their body at all times. During the lactation period test, the person faced the cow at all times for safety reasons, but during the other tests the person looked at the floor, avoiding eye contact with the animal. Behaviours recorded were latency to first touch the human, total time spent interacting with the human (sniffing, licking, touching, playing), time spent looking at human, and the number of withdrawals, bucking and locomotory play events.

For the novel object test, animals entered the arena individually for 15 min where an object was placed at the center of the arena (for the pre- and post-weaning periods a black, 130 L bucket was used; for the puberty period a blue, 130 L bucket was used; and, for the lactation period a camping tent measuring 2.1 x 1.8 m was used). Behaviours recorded were identical to those described for the novel human test.

***Statistical analysis***

All analyses were performed with SAS (version 9.4; SAS Inst. Inc., Cary, NC) using individual animal as the experimental unit. Response variables were summarized by animal for each test and period, and presented as percentage of test time. Data were scrutinized using the UNIVARIATE procedure and probability distribution plots in SAS. To determine the relationship between individual behavioural measures in each test across periods, Spearman rank correlations were performed due to the non-parametric nature of most variables.

A principal component analysis (PCA) was used to condense correlated behavioural measures in the novel environment, novel human and novel object tests into principal components (factors) at each period (i.e. a separate PCA was performed for each period). Prior to analysis, variables were transformed to achieve normality as required (using log_10_ or square root transformations). Frequencies of bucking and withdrawals were too few to be meaningfully included in the analysis. Final input variables from the novel human and object tests included: latency to touch the human and object, time spent touching the human and object, time spent attentive (looking at the human and object) and inattentive, and number of object play events. Final input variables from the novel environment test included time spent exploring, active and inactive. PCA analysis criteria and calculations followed the recommendations outlined by Comrey and Lee [3] and Budaev [4]. For each PCA, the correlation matrix was computed (see Supplementary Tables S4-7) and the first two factors were retained (eigenvalues > 1 and following scree plot examination) and subjected to orthogonal (varimax) rotation. The variables used for analysis met the criteria of Kaiser–Meyer–Olkin measure of sampling adequacy (Pre-weaning = 0.72, Post-weaning = 0.67, Puberty = 0.68, Lactation = 0.47) required for conducting PCA. Communality estimates were adequate for most variables; a variable that was *h^2^* < 0.40 in one of the periods was *h^2^* > 0.40 in another period and thus this variable was retained in the PCA for all periods (Pre-weaning > 0.41, Post-weaning > 0.28, Puberty > 0.01, Lactation > 0.34). Individual scores on each of the PCA factors were extracted using the regression method. A variable was considered to have a high loading on a factor if > ± 0.62.

Pearson correlations were performed between the factor loadings at each period to determine if the correlational structure changed over time (i.e. structural consistency). Tucker’s coefficient of congruence was also performed as a measure of factor similarity (and thus structural consistency) across periods; values between 0.85 to 0.94 indicate high similarity, and values > 0.94 indicate factors can be considered equal (following [5]).

To determine if male and female calves differed in their expression of personality traits (i.e. individual factor loadings), we conducted two general linear models (PROC GLM) where factor loading at each period was the response variable (pre-weaning and post-weaning periods only, since males were only tested at these periods) and sex and birth weight were the explanatory variables. To determine if male and female calves differed in their consistency of personality traits from pre-weaning to post-weaning, a general linear model was conducted (PROC GLM) where post-weaning factor loading was the response variable, and pre-weaning factor loading, sex and birth weight were the explanatory variables.

Data sets and SAS code can be found in the Mendeley repository at: doi: 10.17632/gy7dkst6nm.1

**References**

1. Neave HW, Costa JHC, Weary DM, von Keyserlingk MAG. 2018 Personality is associated with feeding behavior and performance in dairy calves. *J. Dairy Sci.* **101**, 7437–7449. (doi:10.3168/jds.2017-14248)

2. Van Reenen CG, Engel B, Ruis-Heutinck LFM, Van Der Werf JTN, Buist WG, Jones RB, Blokhuis HJ. 2004 Behavioural reactivity of heifer calves in potentially alarming test situations: A multivariate and correlational analysis. *Appl. Anim. Behav. Sci.* **85**, 11–30. (doi:10.1016/j.applanim.2003.09.007)

3. Comrey A., Lee H. 2013 *A first course in factor analysis*. 2nd edn. Psychology Press. (doi:10.1360/zd-2013-43-6-1064)

4. Budaev S V. 2010 Using principal components and factor analysis in animal behaviour research: Caveats and guidelines. *Ethology* **116**, 472–480. (doi:10.1111/j.1439-0310.2010.01758.x)

5. Lorenzo-Seva U, Ten Berge JMF. 2006 Tucker’s Congruence Coefficient as a Meaningful Index of Factor Similarity. *Hogrefe Huber Publ. Methodol.* **2**, 57–64. (doi:10.1027/1614-1881.2.2.57)

**Supplementary Table S1.** Ethogram of behaviours scored during each of the novelty tests when Holstein dairy cattle from two cohorts were tested at 4 developmental periods: pre-weaning (n = 56), post-weaning (n = 89), puberty (n = 30) and lactation (n = 48) (following Neave et al. [1]).

| Behavior | | Description |  |
| --- | --- | --- | --- |
| All Tests | |  |  |
|  | Locomotor Play | Jumping: both forelegs off the ground and extended forwards (number of events)  Running: animal trotting (2 beats) or galloping (3 beats) across or around the enclosure (number of events) |  |
|  | Bucking | Both hind legs are off the ground and extended backwards (number of events) |  |
|  | Resting | Time spent lying down with underside or side of body in full contact with flooring substrate |  |
|  | Withdrawal | Sudden movement backwards (number of events) |  |
| Novel Environment Test | |  |  |
|  | Lick/sniff Walls or Floor | Time spent with muzzle or tongue in contact with either walls or flooring substrate while moving or stationary |  |
|  | Locomotor Activity | Total number of squares crossed with all four feet (test arena divided into 4 equal quadrants) |  |
|  | Inactivity | Time spent standing still without sniffing or licking of walls or floor |  |
| Novel Human and  Novel Object Tests | |  |  |
|  | Latency to Touch | Time until moment animal touches the human or object (muzzle within 5 cm) |  |
|  | Looking Far | Time spent with head oriented toward human or object, more than one body length away |  |
|  | Looking Close | Time spent with head oriented toward human or object, less than one body length away |  |
|  | Far | Time spent with head oriented away from human or object, more than one body length away, or engaged in other behaviors |  |
|  | Touching | Time spent with muzzle in contact with human or object (muzzle within 5 cm) |  |
|  | Object Play | Butting (head in contact with) human or object, or ‘mock butt’ where head is oriented downward and toward but not in contact with human or object |  |

**Supplementary Table S2.** Varimax-rotated loadings on the factors extracted by principal component analysis (PCA) for each developmental period of Holstein dairy cattle (pre-weaning, post-weaning, puberty and lactation). Input variables include the behavioral measures recorded when animals were tested in novel environment, novel human, and novel object tests for 30, 10, and 15 min respectively. Eigenvalues and proportions of total variation explained by each factor are reported. High loadings > ± 0.62 are bolded.

|  | Pre-weaning  (n = 56) | | Post-weaning  (n = 84) | | Puberty  (n = 29) | | Lactation  (n = 47) | |
| --- | --- | --- | --- | --- | --- | --- | --- | --- |
| Variable | Factor 1 | Factor 2 | Factor 1 | Factor 2 | Factor 1 | Factor 2 | Factor 1 | Factor 2 |
| Latency to touch | **-0.87** | 0.10 | **-0.85** | 0.04 | **-0.75** | -0.27 | **-0.62** | -0.44 |
| Time spent touching | **0.93** | 0.15 | **0.76** | 0.43 | **0.86** | -0.15 | **0.76** | 0.30 |
| Inattentive | **-0.62** | 0.40 | -0.51 | -0.61 | **-0.83** | -0.07 | **-0.83** | 0.28 |
| Attentive | **-0.64** | 0.066 | **-0.83** | 0.15 | -0.44 | -0.41 | 0.12 | **-0.69** |
| Object play events | **0.84** | 0.21 | **0.69** | 0.50 | **0.76** | 0.32 | **0.71** | 0.16 |
| Inactive | 0.015 | 0.23 | -0.09 | **-0.73** | -0.15 | **-0.91** | -0.39 | -0.54 |
| Explore | 0.13 | **0.81** | 0.005 | 0.53 | 0.026 | **0.92** | 0.10 | **0.77** |
| Active  (no. quadrants crossed) | -0.18 | **0.69** | -0.03 | **0.68** | 0.096 | 0.076 | 0.48 | -0.33 |
| Eigenvalues | 3.2 | 1.4 | 3.5 | 1.3 | 3.2 | 1.6 | 2.8 | 1.7 |
| Variance explained (%) | 39.7 | 17.8 | 44.1 | 16.8 | 40.5 | 20.0 | 34.6 | 20.8 |
| Interpretation | Bold | Exploratory, active | Bold | Active | Bold | Exploratory, less inactive | Bold | Exploratory, less attentive |

**Supplementary Table S3.** Behavioral responses (mean ± SD, range) of Holstein dairy cattle when tested individually at 4 developmental periods (pre-weaning, post-weaning, puberty, and lactation) in novel environment, novel human, and novel object tests for 30, 10, and 15 min, respectively.

|  | | Pre-weaning (n = 56) | | Post-weaning (n = 89) | | Puberty (n = 30) | | Lactation (n = 48) | |
| --- | --- | --- | --- | --- | --- | --- | --- | --- | --- |
| Test / Behavior | | Mean ± SD | Range | Mean ± SD | Range | Mean ± SD | Range | Mean ± SD | Range |
| Novel environment test | |  |  |  |  |  |  |  |  |
|  | Exploration (% of test time) | 41.2 ± 14.9 | 13.2 – 72.6 | 45.9 ± 11.7 | 24.3 – 73.4 | 37.5 ± 9.7 | 18.1 – 61.5 | 29.9 ± 8.8 | 9.0 – 50.2 |
|  | Inactivity (% of test time) | 23.9 ± 13.1 | 2.1 – 66.4 | 27.9 ± 15.3 | 7.6 – 65.3 | 33.2 ± 13.7 | 12.1 – 63.8 | 37.2 ± 13.4 | 16.6 – 71.3 |
|  | Locomotory play (no.) | 5.0 ± 6.4 | 0 – 24 | 7.7 ± 7.4 | 0 - 31 | 12.3 ± 7.9 | 2 – 34 | 20.0 ± 8.9 | 3 – 39 |
|  | Active (no. quadrants crossed) | 47.4 ± 23.5 | 11 – 118 | 57.4 ± 29.8 | 7 - 156 | 87.8 ± 28.7 | 44 – 163 | 67.0 ± 28.3 | 21 – 136 |
| Novel human test | |  |  |  |  |  |  |  |  |
|  | Latency to touch (s) | 318.5 ± 254.3 | 6 - 600 | 145.2 ± 204.7 | 3 – 600 | 174.8 ± 223.9 | 10 – 600 | 238.8 ± 230.6 | 5 – 600 |
|  | Attentive (% of test time) | 21.1 ± 11.8 | 2.2 – 63.7 | 17.3 ± 9.7 | 1.8 – 68.7 | 13.8 ± 5.2 | 3.2 – 25.7 | 13.7 ± 5.9 | 2.7 – 25.2 |
|  | Inattentive (% of test time) | 57.2 ± 18.9 | 18.2 – 97.8 | 61.8 ± 17.1 | 15.2 – 88.5 | 67.3 ± 13.4 | 41.0 – 88.0 | 77.3 ± 10.4 | 43.0 – 94.0 |
|  | Touching human (% of test time) | 16.1 ± 20.6 | 0 – 70.3 | 19.1 ± 19.7 | 0.0 – 78.0 | 12.5 ± 14.4 | 0.0 – 46.3 | 4.9 ± 9.0 | 0 – 40.7 |
|  | Locomotor play (no.) | 3.5 ± 6.8 | 0 – 36 | 4.8 ± 6.8 | 0 - 31 | 2.2 ± 4.8 | 0 – 24 | 3.5 ± 5.0 | 0 – 21 |
| Novel object test | |  |  |  |  |  |  |  |  |
|  | Latency to touch (s) | 135.7 ± 217.9 | 3 – 900 | 41.3 ± 117.9 | 2 – 900 | 22.2 ± 30.3 | 3 – 129 | 67.7 ± 143.7 | 2 – 900 |
|  | Attentive (% of test time) | 11.2 ± 6.0 | 3.7 – 40.0 | 8.9 ± 4.5 | 3.2 – 24.6 | 7.0 ± 2.9 | 2.6 – 12.9 | 10.3 ± 6.5 | 2.8 – 34.8 |
|  | Inattentive (% of test time) | 63.2 ± 16.9 | 10.9 – 92.3 | 70.3 ± 13.1 | 39.1 – 91.9 | 66.6 ± 14.8 | 28.0 – 88.1 | 77.1 ± 8.4 | 57.8 – 91.1 |
|  | Touching object (% of test time) | 18.0 ± 13.2 | 0.0 – 55.2 | 18.8 ± 11.5 | 0 – 51.3 | 19.4 ± 13.6 | 4.2 – 61.7 | 8.3 ± 7.0 | 0.0 – 35.0 |
|  | Locomotor play (no.) | 3.6 ± 6.1 | 0 - 27 | 5.6 ± 7.2 | 0 - 38 | 2.7 ± 2.9 | 0 - 12 | 3.7 ± 4.5 | 0 - 17 |

**Supplementary Table S4.** Correlation matrix used in Principal Component Analysis of the pre-weaning developmental period.

| **Correlations** | | | | | | | | |
| --- | --- | --- | --- | --- | --- | --- | --- | --- |
|  | **latency_log10** | **touch** | **far** | **look** | **OPevents_sqrt** | **NE_inactive** | **NE_TotalLick** | **NE_TotalQuad** |
| latency_log10 | 1.00000 | -0.77140 | 0.48092 | 0.48084 | -0.65852 | -0.09624 | 0.03935 | 0.18244 |
| touch | -0.77140 | 1.00000 | -0.47813 | -0.50451 | 0.77936 | 0.04963 | 0.25331 | -0.11755 |
| far | 0.48092 | -0.47813 | 1.00000 | 0.26608 | -0.37576 | 0.17973 | 0.14195 | 0.20237 |
| look | 0.48084 | -0.50451 | 0.26608 | 1.00000 | -0.38885 | 0.17202 | -0.01090 | 0.06375 |
| OPevents_sqrt | -0.65852 | 0.77936 | -0.37576 | -0.38885 | 1.00000 | 0.12161 | 0.19071 | -0.04583 |
| NE_inactive | -0.09624 | 0.04963 | 0.17973 | 0.17202 | 0.12161 | 1.00000 | 0.00306 | -0.01292 |
| NE_TotalLick | 0.03935 | 0.25331 | 0.14195 | -0.01090 | 0.19071 | 0.00306 | 1.00000 | 0.28813 |
| NE_TotalQuad | 0.18244 | -0.11755 | 0.20237 | 0.06375 | -0.04583 | -0.01292 | 0.28813 | 1.00000 |

**Supplementary Table S5.** Correlation matrix used in Principal Component Analysis of the post-weaning developmental period.

| **Correlations** | | | | | | | | |
| --- | --- | --- | --- | --- | --- | --- | --- | --- |
|  | **latency_log10** | **touch** | **far** | **look_log10** | **OPevents_sqrt** | **NE_inactive_log10** | **NE_TotalLick** | **NE_TotalQuad** |
| latency_log10 | 1.00000 | -0.53399 | 0.31387 | 0.62560 | -0.50750 | 0.13209 | -0.13223 | -0.03607 |
| touch | -0.53399 | 1.00000 | -0.88282 | -0.48600 | 0.75342 | -0.27559 | 0.15654 | 0.23478 |
| far | 0.31387 | -0.88282 | 1.00000 | 0.17250 | -0.70311 | 0.36900 | -0.15557 | -0.25777 |
| look_log10 | 0.62560 | -0.48600 | 0.17250 | 1.00000 | -0.48047 | 0.10263 | -0.01540 | -0.07438 |
| OPevents_sqrt | -0.50750 | 0.75342 | -0.70311 | -0.48047 | 1.00000 | -0.38307 | 0.13959 | 0.38064 |
| NE_inactive_log10 | 0.13209 | -0.27559 | 0.36900 | 0.10263 | -0.38307 | 1.00000 | -0.11246 | -0.30182 |
| NE_TotalLick | -0.13223 | 0.15654 | -0.15557 | -0.01540 | 0.13959 | -0.11246 | 1.00000 | 0.17394 |
| NE_TotalQuad | -0.03607 | 0.23478 | -0.25777 | -0.07438 | 0.38064 | -0.30182 | 0.17394 | 1.00000 |

**Supplementary Table S6.** Correlation matrix used in Principal Component Analysis of the puberty developmental period.

| **Correlations** | | | | | | | | |
| --- | --- | --- | --- | --- | --- | --- | --- | --- |
|  | **latency_log10** | **touch_log10** | **far** | **look** | **OPevents_log10** | **NE_inactive** | **NE_TotalLick** | **NE_TotalQuad** |
| latency_log10 | 1.00000 | -0.53158 | 0.58464 | 0.34449 | -0.51404 | 0.39186 | -0.22482 | 0.00286 |
| touch_log10 | -0.53158 | 1.00000 | -0.57581 | -0.29917 | 0.53061 | -0.00931 | -0.03331 | 0.14788 |
| far | 0.58464 | -0.57581 | 1.00000 | 0.24040 | -0.62237 | 0.25290 | -0.09562 | 0.06468 |
| look | 0.34449 | -0.29917 | 0.24040 | 1.00000 | -0.39967 | 0.33173 | -0.26414 | -0.08439 |
| OPevents_log10 | -0.51404 | 0.53061 | -0.62237 | -0.39967 | 1.00000 | -0.31827 | 0.34122 | 0.14133 |
| NE_inactive | 0.39186 | -0.00931 | 0.25290 | 0.33173 | -0.31827 | 1.00000 | -0.74538 | -0.07169 |
| NE_TotalLick | -0.22482 | -0.03331 | -0.09562 | -0.26414 | 0.34122 | -0.74538 | 1.00000 | 0.03454 |
| NE_TotalQuad | 0.00286 | 0.14788 | 0.06468 | -0.08439 | 0.14133 | -0.07169 | 0.03454 | 1.00000 |

**Supplementary Table S7.** Correlation matrix used in Principal Component Analysis of the lactation developmental period.

| **Correlations** | | | | | | | | |
| --- | --- | --- | --- | --- | --- | --- | --- | --- |
|  | **latency_sqrt** | **touch_log10** | **far** | **look** | **OPevents_log10** | **NE_inactive** | **NE_TotalLick** | **NE_TotalQuad** |
| latency_sqrt | 1.00000 | -0.54190 | 0.35608 | 0.31742 | -0.43610 | 0.27998 | -0.21783 | -0.09473 |
| touch_log10 | -0.54190 | 1.00000 | -0.64114 | -0.22194 | 0.53036 | -0.19493 | 0.24791 | 0.01616 |
| far | 0.35608 | -0.64114 | 1.00000 | -0.39296 | -0.37462 | 0.11515 | 0.01381 | -0.24706 |
| look | 0.31742 | -0.22194 | -0.39296 | 1.00000 | -0.22008 | 0.01009 | -0.13709 | 0.23422 |
| OPevents_log10 | -0.43610 | 0.53036 | -0.37462 | -0.22008 | 1.00000 | -0.25025 | -0.00483 | 0.27089 |
| NE_inactive | 0.27998 | -0.19493 | 0.11515 | 0.01009 | -0.25025 | 1.00000 | -0.71081 | -0.34629 |
| NE_TotalLick | -0.21783 | 0.24791 | 0.01381 | -0.13709 | -0.00483 | -0.71081 | 1.00000 | -0.16833 |
| NE_TotalQuad | -0.09473 | 0.01616 | -0.24706 | 0.23422 | 0.27089 | -0.34629 | -0.16833 | 1.00000 |
